# The Edmonton frail scale: a feasibility study on assessing frailty among older adults with multimorbidity in Norwegian primary health care

**DOI:** 10.1186/s12875-025-02996-7

**Published:** 2025-09-26

**Authors:** Turid Rimereit Aarønes, Kristin Taraldsen, Are Hugo Pripp, Linda Aimée Hartford Kvæl

**Affiliations:** 1 Faculty of Health Sciences, Department of Rehabilitation Science and Health Technology, OsloMet – Oslo Metropolitan University, Oslo, Norway; 2 Department for Research, Innovation, Education, and Health Service Development, Møre og Romsdal Hospital Trust, Ålesund, Norway; 3 Health Services Research Unit (HØKH), Akershus University Hospital, P.O. Box 1000, Lørenskog, 1478 Norway; 4 Research Centre for Habilitation and Rehabilitation Models and Services (CHARM), Institute of Health and Society, Faculty of Medicine, University of Oslo, Oslo, Norway

**Keywords:** Edmonton frail scale, Frailty assessment, Multimorbidity management, Primary healthcare, Older adults, Healthcare professionals, Person-centred care, Structured assessments, Feasibility study, Norway healthcare integration

## Abstract

**Background:**

The growing prevalence of multimorbidity and frailty, driven by an ageing population and changing health trends, is placing significant pressure on healthcare systems. Frailty assessments provide valuable insights into patient vulnerability, allowing for early interventions to prevent functional decline and reduce hospitalisations. Despite their importance, standardised frailty assessment instruments are not widely used in primary care. This study investigated the feasibility of using one such instrument, the multidimensional Edmonton Frail Scale (EFS), in Norwegian primary healthcare.

**Methods:**

This feasibility study involved 14 healthcare professionals (10 physiotherapists and four nurses) from primary healthcare in three Norwegian municipalities. Participants were trained to use the EFS to assess and generate frailty scores. Four focus group interviews explored these professionals’ experiences of using the EFS with home-dwelling older adults with multimorbidity. The EFS scores were analysed with descriptive statistics, and the interview data underwent reflexive thematic analysis.

**Results:**

Through interview analysis, we identified three main themes: (i) enabling collaborative planning, (ii) facilitating comprehensive assessments, and (iii) integrating and understanding EFS competently. The assessment of frailty using the EFS among home-dwelling older adults with multimorbidity (*n* = 86) revealed scores ranging from 2 to 14, with 2% of these adults categorised as fit, 18% as pre-frail and 80% as frail. Most participants failed the clock test, and many had been hospitalised in the past year. Despite these challenges, 83% reported very good or fair self-perceived health, though the EFS scores indicated significant dependency in daily activities. Polypharmacy was common, with three-quarters of patients taking five or more medications. Additionally, recent weight loss, mobility issues and sadness or depression were frequently reported.

**Conclusions:**

The EFS supported collaborative care planning by identifying frailty domains, facilitating tailored interventions to address challenges such as polypharmacy, mobility issues, emotional well-being, and dependency in daily activities. The themes of collaborative care, comprehensive assessments, and competent integration highlight the EFS’s potential as a multidimensional instrument for routine use in primary care. With proper healthcare professional training, the EFS can promote person-centred care, improve overall care quality and support the early detection and prevention of complications, addressing the complex needs of older adults with multimorbidity.

**Supplementary Information:**

The online version contains supplementary material available at 10.1186/s12875-025-02996-7.

## Background

The global population is ageing, leading to a growing prevalence of multimorbidity, often accompanied by frailty [1, 2]. Together, these conditions place increasing pressure on healthcare systems [3, 4], as exacerbated by advancements in medical technology, rising healthcare costs and heightened expectations for quality care [1]. Multimorbidity refers to the presence of two or more long-term health conditions [3], while frailty often accompanies multimorbidity and involves diminished bodily functions [5]. Both conditions are associated with heightened risks of functional decline, hospitalisations and other adverse outcomes [6, 7]. Managing older adults with multimorbidity is complex, requiring attending to multiple chronic conditions while acknowledging patients’ preferences [8]. This necessitates an integrated care approach, where healthcare professionals (HCPs) collaborate across disciplines to deliver more person-centred care [9].

Assessing individuals with multimorbidity for frailty is a recommended practice to identify functional decline and enable proactive, holistic care actions to mitigate risks such as hospitalisations, medication errors, social isolation and nutritional deficiencies [9, 10]. This approach supports comprehensive care tailored to the complex needs of older adults with multimorbidity. Thus, timely frailty management can improve quality of life for older adults and reduce the burden on the healthcare system [11, 12].

Norway, like many European countries, has shifted towards more outpatient care and has a well-developed long-term care system, designed to support individuals with chronic illnesses, disabilities or age-related needs over an extended period [13]. This system’s primary healthcare services include home care and rehabilitation tailored to individual needs, with a focus on restoring function and independence for individuals with varying abilities, including those with multimorbidity [14, 15]. However, despite offering a wide range of services for home-dwelling older adults with complex care needs, Norway faces challenges in achieving continuity and coordination in primary care compared to other European nations [13]. Initiatives such as primary healthcare teams have been piloted in some municipalities to strengthen care coordination, continuity and patient assessment [13]. These efforts are critical, as HCPs in primary care often face resource constraints, limiting time for comprehensive assessments and interprofessional communication [16]. An integrated approach is thus essential to address patients’ changing needs effectively and deliver tailored interventions [17].

Research shows that frailty assessment practices in Norwegian primary care often rely on clinical expertise, observational insights, and informal discussions among HCPs [18, 19]. Therefore, these practices could benefit from greater systematisation and standardisation to ensure consistency, foster more structured services and reduce fragmentation across professions and care levels [20]. In addition, the lack of a unified and widespread frailty instrument leads to uncoordinated evaluations that fail to capture the comprehensive health status of older adults, highlighting the urgent need for a structured and more uniform approach to frailty assessment [21, 22]. Standardised instruments enhance assessment quality by ensuring consistency, improving reliability and providing a structured framework for evaluating frailty. They reduce reliance on individual expertise and allow for repeated measures, enabling HCPs to adapt interventions over time [21, 23]. Despite the availability of numerous instruments, inconsistent use and the lack of a gold standard hinders the integration of frailty assessments into routine practice [24].

The Edmonton Frail Scale (EFS) is a user-friendly, validated instrument that assesses frailty in older adults across multiple domains, including cognition, nutrition, physical function and social support [22, 24, 25]. The EFS was selected because it provides clinically interpretable frailty assessments that can help HCPs identify specific areas of concern and facilitate discussions with patients [25]. Its multidimensional structure supports a holistic view of the patient, making it useful for comprehensive evaluations and fostering collaboration across healthcare teams. Its quick and practical design makes it suitable for time-constrained settings, offering actionable frailty scores to guide person-centred care [26]. In comparison, tools like the Complex Needs Case-finding Tool-6 (CONECT-6) [27] and the Person Centred Assessment Method (PCAM) [28] focus on broader health and social needs without providing specific frailty scores, while others, such as the Frailty Index [29], Tilburg Frailty Indicator (TFI) [30] and Fried Frailty Criteria [5], are either time-intensive, rely solely on self-report, or exclude key dimensions. The EFS, therefore, appears well suited for exploring tailored interventions and improving care coordination in the primary care context. The EFS has demonstrated feasibility in various international contexts, such as outpatient clinics [31], hospitals [32–38] and primary care settings [26, 39, 40]. However, its use for home-dwelling older adults with multimorbidity in Norwegian primary healthcare remains unexplored. Little is known about how HCPs perceive the feasibility and usability of the EFS in home care as well. The EFS can potentially support individualised care by identifying specific areas requiring intervention, which can help HCPs so they can deliver more continuous and tailored care [16, 17, 24].

Given Norway’s focus on strengthening care coordination and assessment in primary healthcare, the EFS offers a structured approach to address these priorities. However, research is needed to evaluate the EFS’s practical utility, acceptance among HCPs and compatibility with existing workflows in Norwegian primary care settings [41]. This study aimed to address these gaps by investigating the feasibility of using the EFS in Norwegian primary healthcare, providing insights into its potential integration and its impact on improving multimorbidity management and healthcare delivery.

## Methods

### Design

**Fig. 1:**
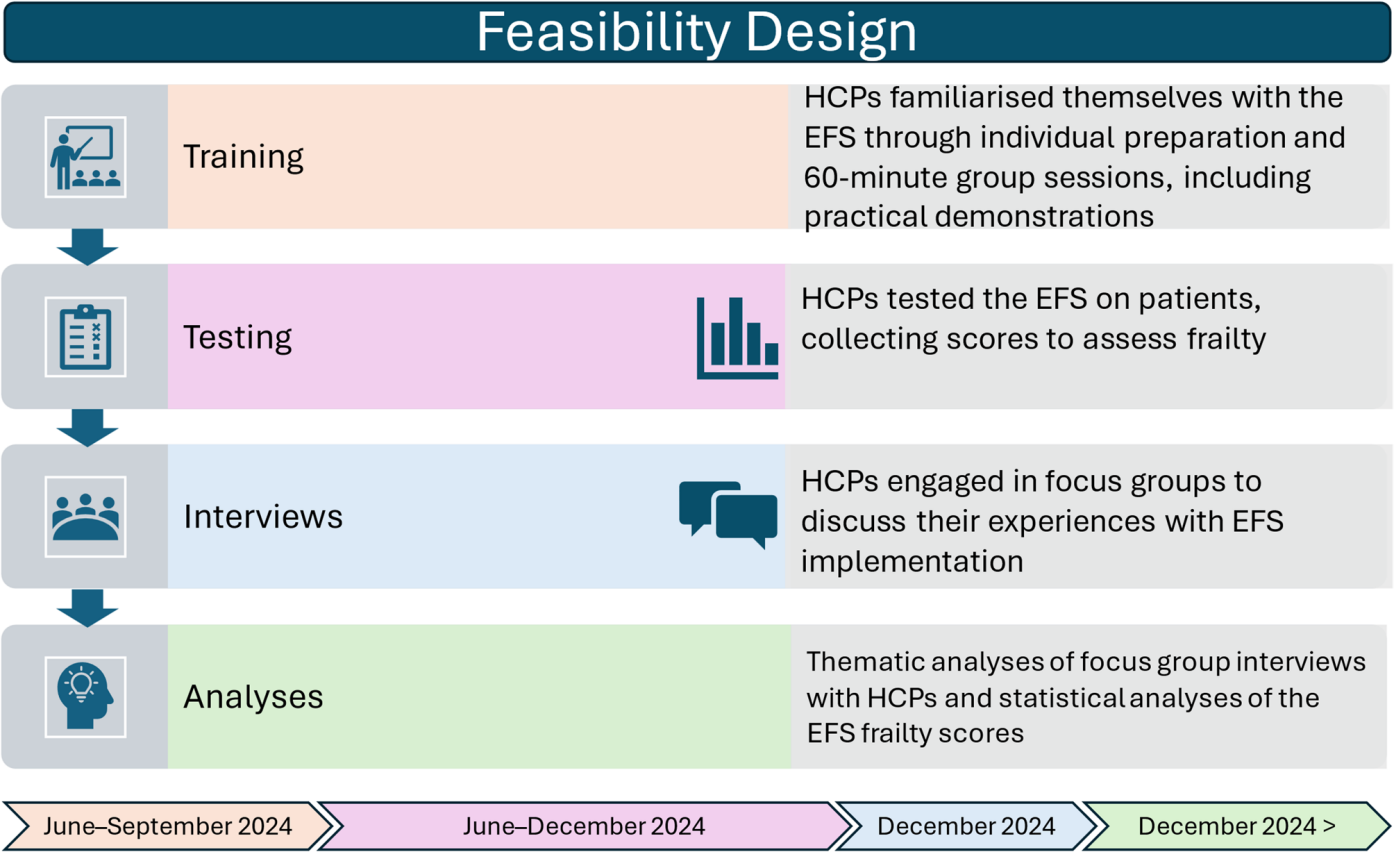
Feasibility design overview. An overview of the study design, which consists of four parts: Training, testing the Edmonton Frail Scale (EFS), conducting interviews with healthcare professionals (HCPs) and analyses

Feasibility of the EFS was assessed by (i) qualitative interviews with HCPs to explore their experiences of using the EFS in primary care, and (ii) quantitative analysis of frailty scores to describe and characterise frailty among older multimorbid patients in a primary care setting. Initially, the HCPs received a training package to familiarise themselves with the EFS, then tested the instrument with home-dwelling older adults experiencing multimorbidity in primary healthcare. Focus groups were chosen to facilitate dynamic discussions, allowing participants to share experiences, build on each other’s reflections and explore shared challenges and opportunities in using the EFS [42]. This approach allowed participants to reflect on collective experiences and the team-based integration of the tool. The EFS data were collected to provide contextual information about the patient population for whom the instrument was used and to ground the subsequent HCP interviews in the EFS application. Additionally, the EFS data served an evaluative function by allowing for an exploration of patient characteristics, scoring distribution, and a comparison with findings in existing clinical literature. The study started recruitment in March 2024, with data collection taking place from June to December 2024. Figure 1 provides an overview of the feasibility design.

### Study setting

The study was conducted within the primary Healthcare systems of three municipalities in one region in Norway. These municipalities were chosen to reflect variations in size, ranging from 13,000 to 67,000 inhabitants, and geographical distribution, to capture diverse healthcare practices and patient demographics, which are important for assessing the feasibility of the EFS in different settings. In Norway, municipalities are responsible for managing primary care and social services, which are provided by general practitioners, nursing homes, home-based services and rehabilitation services [13]. Rehabilitation services include short-term stays at intermediate care institutions and reablement in older adults’ homes.

### The Edmonton Frail Scale

The EFS is a validated instrument for measuring frailty, tested across various cultural contexts to produce reliable results and accurately detect changes in frailty [24]. It is suitable for use by non-geriatricians in both community and inpatient settings, offering capabilities like pre-frailty verification and mortality prediction [24, 25]. By identifying frailty levels and associated health concerns, the EFS helps HCPs tailor care to individual needs, including interventions such as promoting physical activity, providing nutritional support, enhancing social networks and reducing polypharmacy [43].

The EFS covers nine domains of frailty: cognition, general Health status, functional independence, social support, medication use, nutrition, mood, continence and functional performance. Of the 11 items in the EFS, two are performance-based (the Clock Test and the Timed Up and Go Test), while the rest are answered by the interviewee. Scores range from 0 to 17, with higher scores indicating greater frailty [25]. Our study was intended to inform decisions about integrating the EFS into routine practice, focusing on its feasibility in Norwegian primary healthcare settings.

### Recruitment and participants

To initiate participant recruitment, we sent information letters by email to healthcare unit managers, who discussed participation with their teams and provided the contact details of potential HCP participants or individuals who expressed their interest directly. We strategically selected HCPs experienced in working with older home-dwelling individuals with multimorbidity using the following inclusion criteria: preferably experienced with multidisciplinary teamwork and patient assessment; must have worked in relevant setting ≥ 1 year; and hold at least 50% of a full-time position. HCPs who were not able to communicate adequately in Norwegian were excluded. All participants received information letters and signed consent forms prior to joining the study.

Among the 14 HCPs recruited, 10 were physiotherapists and four were nurses, and comprised 12 women and two men. These participants worked across diverse Healthcare settings, including rehabilitation and reablement teams, physiotherapy services, short-term facilities, outpatient clinics, hospital wards and coordination offices. The participants were a mean age of 43.5 years old (ranging from 30 to 55 years) and had an average of six years of experience in their current roles.

The HCPs were tasked with recruiting approximately 10 eligible older adult patients with multimorbidity each from their existing patient pools. To support this process, they were provided with information letters to distribute to potential participants. Only patients who signed the consent forms were enrolled in the study. The selection criteria for older adults with multimorbidity included being home-dwelling, 65 years or older, having two or more chronic conditions, and receiving municipal services such as home-nursing or rehabilitation. Exclusion criteria included pronounced cognitive impairment, dementia, severe psychiatric conditions and inability to communicate in Norwegian. In total, 86 older adults with multimorbidity were recruited, providing ample data on frailty levels.

### Data collection

The HCPs underwent EFS training led by the certified first author. They received a training video and toolkit via email, licensed from the EFS developers [44]. One week later, 60-minute group sessions were held at their workplaces, featuring practical demonstrations of the Norwegian version of the EFS. Equipment and support sheets were provided to ensure the EFS’s correct use and standardisation. As the HCPs were new to the EFS, some variability in scoring was anticipated. To address this, the first author reviewed and, where needed, revised clock test scorings to ensure consistency, maintain uniformity in the application of the tool, and enhance the accuracy of the data.

The HCPs assessed the recruited patients using the EFS either at the patients’ homes or within primary care facilities, selecting the most practical and acceptable location for each individual. Numerical scores from these assessments were securely stored and collected by the first author on the day of the focus group interviews. The HCPs collected and verified patient information from journals, including gender, age, education level, municipality, marital status, number of diagnoses, home services and use of walking aids. Throughout the testing period, the first author provided support to the HCPs and monitored their progress.

We then developed a semi-structured interview guide to explore the HCPs’ experiences with the EFS, focusing on its applicability, integration into workflows and potential for routine use. Input from a reference group consisting of a user representative, HCP and professor helped refine the guide (see Additional File 1). HCP background data, such as gender, age, education, profession, workplace and years of experience, were collected by the first author. The combination of qualitative and quantitative data provided a comprehensive evaluation of the EFS’s feasibility.

The four focus group interviews were conducted at or near the HCPs’ workplaces, with participants divided into one group of two, two groups of three, and one group of four. Two HCPs were unable to participate due to Health-related reasons. The interviews were conducted on two consecutive days in December 2024. All interviews were held in person in a secluded room with a welcoming atmosphere and were recorded as MP3 files. The first author facilitated the discussions, while the third author acted as an observer. Each interview lasted between 60 and 75 min.

We concluded that our study had sufficient information power after conducting four focus groups interviews, supported by a representative sample, strong dialogues, and the integration of both qualitative and quantitative research methods to explore our focused aims [45]. In line with Malterud et al. [45], we considered the specificity of the research question, the diversity and expertise of participants, and the depth of the data collected, ensuring that the data were rich and sufficient to address the study’s aims.

Although the quantitative EFS data were collected before the focus group interviews, the qualitative analysis and findings are presented first in both the analysis and results sections of this study. This reflects the primary aim of exploring the feasibility and relevance of using the EFS from the perspective of HCPs.

### Data analysis

All interviews were analysed using Braun and Clarke’s six-step reflexive thematic analysis [46]. The first author thoroughly transcribed the interviews verbatim before engaging the fourth author in a review of the transcripts to identify core findings. All transcripts were closely coded by the first author, who subsequently developed potential initial themes from these codes (see Table 1). In reflexive thematic analysis, the practice of single-person coding is both standard and effective, reinforcing the depth and reflexivity of the analysis [46]. Through an iterative analysis process, nine initial themes were identified and refined from the dataset in discussions with the fourth author. In developing these themes, we were inspired by COSMIN (Consensus-Based Standards for the Selection of Health Measurement Instruments) criteria for content validity – comprehensiveness, relevance, and comprehensibility – to guide our analysis and ensure that the themes accurately captured the nuances of the data [47]. These initial themes were organised into potential main themes and discussed among the authors to ensure they comprehensively reflected the HCPs’ thoughts and experiences with the EFS. The software tool HyperResearch was utilised to facilitate the analysis process [48].

**Table 1:** Example of transitioning from quote to code and initial theme

Quote	Code label	Initial theme
“Yes, for those patients I knew from before, the results were less surprising. However, for new patients, the insights could be more unexpected. They might seem very upbeat during previous telephone contact or [at the] first meeting. Then we discussed the score, especially the score on the clock test, and also their independence in daily activities. Many receive help, but it is often camouflaged, as it comes from relatives rather than home care or other services.” (HCP3, Physiotherapist)	Unexpected cognitive insights from clock test	The benefits of a multidimensional instrument
“Yes, I think what’s nice about the test is that it covers many variables and areas, including cognition, physical function, nutrition and medications. It touches on many different aspects, which I believe is positive for assessing and understanding the patient more comprehensively.” (HCP4, Physiotherapist)	Understanding the patient comprehensively	

The EFS scores were analysed using descriptive statistics, including frequencies, percentages and medians. Two questions involved function tests, while the remaining questions relied on self-reported answers collected by the HCPs. The substantial number of completed tests offered rich data on the patients’ frailty levels and insights into all health domains covered by the EFS.

## Results

### Feasibility insights from focus group interviews with HCPs

Our interview analysis identified three main themes that illustrate the multifaceted impact of the EFS in primary healthcare settings. First, the theme “enabling collaborative planning” underscores the EFS’s role in enhancing coordination and communication among HCPs. Second, the theme “facilitating comprehensive assessments” highlights the EFS’s ability to provide thorough and multidimensional evaluations. Finally, the theme “integrating and understanding EFS competently” focuses on the practical aspects of using the EFS, including its adaptability, training requirements and efficient integration into clinical workflows. The identified themes are listed in Table 2.

**Table 2:** Mapping initial themes to main themes

Initial themes	Main themes
Filling a gap in healthcare practice with standardised assessments	Enabling collaborative planning
Boundary object for collaboration	
Assessing needs of new patients	
The benefits of a multidimensional instrument	Facilitating comprehensive assessments
Confidence in the comprehensive patient profile	
Frailty score in service negotiation	
Versatile across groups and settings	Integrating and understanding EFS competently
Training for effective use	
Efficient integration in clinical workflows	

### Enabling collaborative planning

This first theme highlights HCPs` views on the relevance of the EFS in addressing a gap in healthcare practice by providing standardised, objective assessments that enhance care coordination and foster discussions across health teams and levels, particularly for new patients with limited prior interaction with healthcare services.

Several HCPs mentioned that the EFS was an instrument that had been missing in their practice and had the potential to fill a gap where no frailty screening instruments had been used before. As one nurse noted about their home nursing service, *“No*,* they don’t use any [frailty screening instrument]. At least not with us”* (HCP12, Nurse). Other HCPs observed that the EFS provided a more standardised and objective approach to assessments compared to their usual practice. Typically, HCPs relied on subjective assessments based on their clinical judgement, which could lead to inconsistencies and a fragmented view of patient needs. They highlighted this as a positive feature of the EFS, emphasising its relevance in enhancing assessment quality by securing consistency. As one participant noted:

*“I think structured assessment is very underestimated. We often place too much faith in our own judgement. If we used instruments like the EFS*,* we would have [a] much more consistent starting point with each individual patient”* (HCP12, Nurse).

Some HCPs reported that the EFS had the potential to enhance collaboration amongst themselves, both within teams as well as across organisational boundaries. Several HCPs mentioned that the EFS could serve as a focal point for discussions, considering its various domains and the overall frailty score. By facilitating communication and shared understanding of frailty scores and implications, the EFS emphasised its relevance in promoting interdisciplinary, shared language, and collaboration towards care and interventions more tailored to individual needs. As one participant noted:

*“I would say this is an assessment instrument that has been missing. With the population ageing*,* there is an increasing need for interaction*,* interdisciplinarity and a common language. I believe the EFS can help us achieve better interaction*,* whether interdepartmentally or within a department”* (HCP11, Physiotherapist).

Many HCPs also found the EFS efficient for assessing patients new to primary healthcare services. These patients often had minimal prior use of services and were considered to have slipped under the radar, suggesting their health needs could have been addressed earlier. HCPs believed early frailty detection through the EFS was crucial, emphasising its relevance in identifying patients with unmet needs and supporting early interventions to help these patients remain at home longer and manage their multimorbidity with appropriate, and potentially reduced, assistance:

*“But there are cases where I think it could be useful to point out that a patient is actually on the verge of being seriously frail*,* even though she has gone a little under the radar. It [the EFS] could be used for such patients”* (HCP2, Physiotherapist).

This theme illustrates the EFS’s relevance in fostering communication and coordination among HCPs, facilitating better care planning and interventions tailored to individual needs.

### Facilitating comprehensive assessments

The second theme highlights how the EFS offered a holistic evaluation by covering multiple dimensions of patient health, including cognitive, physical and self-reported aspects. The HCPs emphasised the importance of trusting the complete and comprehensive picture provided by the EFS, which could be used to advocate for necessary services.

The HCPs consistently noted that the EFS’ multidimensional approach provided a more comprehensive health picture than their usual assessments. While the EFS included their typical areas of assessment, it also provided additional information, such as cognitive insights and issues not typically addressed in initial assessments, like incontinence, mental health or social support needs. This approach reflects the EFS’s comprehensiveness, valued by HCPs for capturing overlooked aspects. As one participant said:

*“There are components in the EFS that I don’t know of in any other screenings. It includes aspects like nutrition*,* mood*,* whether you feel sad or depressed*,* and social factors. It provides a comprehensive view of the whole person*,* which I think is very good.”* (HCP9, Physiotherapist).

Several HCPs acknowledged the importance of trusting the comprehensive health picture provided by the EFS, even when faced with uncertainties in self-reported answers, particularly following unexpected failures in the clock test. Despite these initial doubts, the HCPs ultimately relied on the EFS scores to guide their assessments, illustrating how the EFS’s comprehensiveness supported HCPs in decision-making. As one HCP noted, patients who struggled with the clock test often doubted their own memory regarding medications they take, underscoring the necessity of relying on the comprehensive EFS evaluation:

*“Yes*,* and then they don’t completely trust their own memory regarding their medications. They might say*,* ‘Now I’m not quite sure what medications I’m taking’ right? If they had a positive experience at the beginning*,* they would likely have said*,* ‘Yes*,* I’m taking five medications’ with greater self-confidence”* (HCP7, Physiotherapist).

The HCPs valued the EFS for its rapid screening capabilities and score, which proved useful in clinical practice for monitoring and comparing frailty levels. This comprehensiveness provided a practical and multidimensional overview of patients’ health and functional level, allowing comparisons to previous scores and helping HCPs determining the best course of action. Additionally, some HCPs mentioned that the EFS could be used to negotiate health services with the allocation office on behalf of the patient. One participant noted:

*“You can’t focus blindly on just one aspect: It’s about the whole picture – it’s the total evaluation. We need to follow up on these patients eventually*,* including with a physiotherapist. If this form is used by the allocation office*,* it can serve as an instrument to determine what services to refer them to”* (HCP9, Physiotherapist).

This theme illustrates the EFS’s comprehensiveness in providing a multidimensional evaluation of frailty, offering HCPs a thorough understanding of patients’ health and functional levels, while equipping them with structured and actionable insights to support informed decision-making and tailored interventions. It also emphasises the EFS’s utility in clinical practice for monitoring frailty over time and advocating for necessary services.

### Integrating and understanding EFS competently

This final theme highlights the adaptability of the EFS across professional groups and healthcare settings, emphasising the need for training to ensure competent use and leveraging of the EFS`s comprehensibility for effective application. It also underscores the importance of integrating the EFS into existing workflows to enhance its practicality.

Several HCPs described a service with limited resources managing an increasing number of older patients. In this context, they stated that the EFS should ideally be implemented with minimal additional workload, further underscoring its comprehensibility as a tool that is straightforward and easy to integrate into daily practice. Additionally, the HCPs emphasised that the EFS could potentially be used by various professional groups across primary healthcare services. By employing the same frailty screening instrument, they noted that results could be compared to enhance care coordination and joint efforts. As one HCP said:

*“I think it’s [the EFS] a test that isn’t specific to physiotherapy; it could be used by different professional groups. Definitely in home care*,* and not just by nurses*,* but also by social workers and other health professionals. It could be used in nursing homes*,* and maybe even at day centres.”* (HCP4, Physiotherapist).

The HCPs further emphasised the importance of hands-on training, especially for unfamiliar components of the EFS like the clock test. They viewed this as essential for ensuring confidence and competence in using the instrument effectively, while also stressing the importance of the EFS being comprehensible for all users. The HCPs also raised the importance of framing questions competently to ensure that patients understood them and felt comfortable, especially for the clock test and issues of a more sensitive nature. As one participant noted:

*“In my mind*,* if you’re going to do a screening*,* you’d want to go through it quickly and identify the next steps. But then it becomes natural to spend some time on it. For me*,* it comes completely naturally.”* (HCP7, Physiotherapist).

Additionally, HCPs found training necessary to administering the EFS and accurately scoring the results. As one participant stated:

*“Yes*,* the clock test was new to me*,* and I found it the most difficult to perform in practice. This may be because I have no prior experience with clock tests. Additionally*,* it was a different type of clock test than what our occupational therapists use*,* and they have different assessment criteria. I found it challenging to assess the criteria in that test”* (HCP8, Physiotherapist).

Many HCPs described the EFS as most valuable when seamlessly integrated into their workflows. They considered logistical factors, such as deciding which existing tests to replace with the EFS and determining the optimal sequence for asking the EFS questions. With experience, HCPs found the EFS easier to use, facilitating its integration into workflows and allowing for adjustments in questioning based on patient responses. This ease of use and adaptability further reflects the EFS’s high level of comprehensibility. One participant noted:

*“Yes*,* I think that as you become familiar with the instrument and use it regularly*,* you get better at using it*,* just like with any other instrument. You can then adjust to the questions and come up with follow-up questions along the way. It’s easy to do because the EFS provides a very straightforward starting point”.* (HCP12, Nurse)

This theme emphasises that with proper training and integration into workflows, the EFS’s comprehensibility can make it a valuable instrument for professional groups, improving the consistency and quality of frailty assessments in individuals with multimorbidity.

### EFS scores in older adults with multimorbidity

The 86 patients who participated in the study (see Table 3) were home-dwelling older adults with multimorbidity, with a mean age of 83 years (ranging from 71 to 95 years). Of these, 63% (*n* = 54) were women. On average, the patients had 4.5 chronic diagnoses each (ranging from two to 13) and received two municipal home health services (ranging from one to six). Approximately one-third had attended higher education, though this varied across municipalities: Only 8% of patients in the smallest municipality had high education levels, compared to 39% and 40% in the other two. In terms of living arrangements, 55% (*n* = 47) lived alone.

**Table 3:** Characteristics of older adult participants (*n* = 86)

Characteristics	*n* (%)
Gender	
Female	54 (63)
Male	32 (37)
Age (years)	
71–80	24 (28)
81–90	54 (63)
91–95	8 (9)
Average	83.5
Education level (Low = primary or lower secondary school. High = upper secondary school or college/university)	
Low	60 (70)
High	26 (30)
Walking aids	
Yes	71 (83)
No	15 (17)
Living arrangements	
Lives alone	47 (55)
Cohabiting with partner/family	39 (45)
Municipality	
A – second largest	18 (20)
B – largest	43 (50)
C – smallest	25 (30)

The EFS provided a detailed overview of frailty levels among the 86 older adults. Frailty scores were distributed across five categories: fit (*n* = 2), vulnerable (*n* = 15), mild (*n* = 21), moderate (*n* = 30), and severe (*n* = 18), with scores ranging from 2 to 14, illustrating a broad spectrum of frailty. These results demonstrate the EFS’s ability to capture the diverse frailty levels within this population, providing actionable insights for tailoring interventions in primary healthcare and may highlight its feasibility in effectively assessing and stratifying older adults with multimorbidity in primary care settings.

**Fig. 2:**
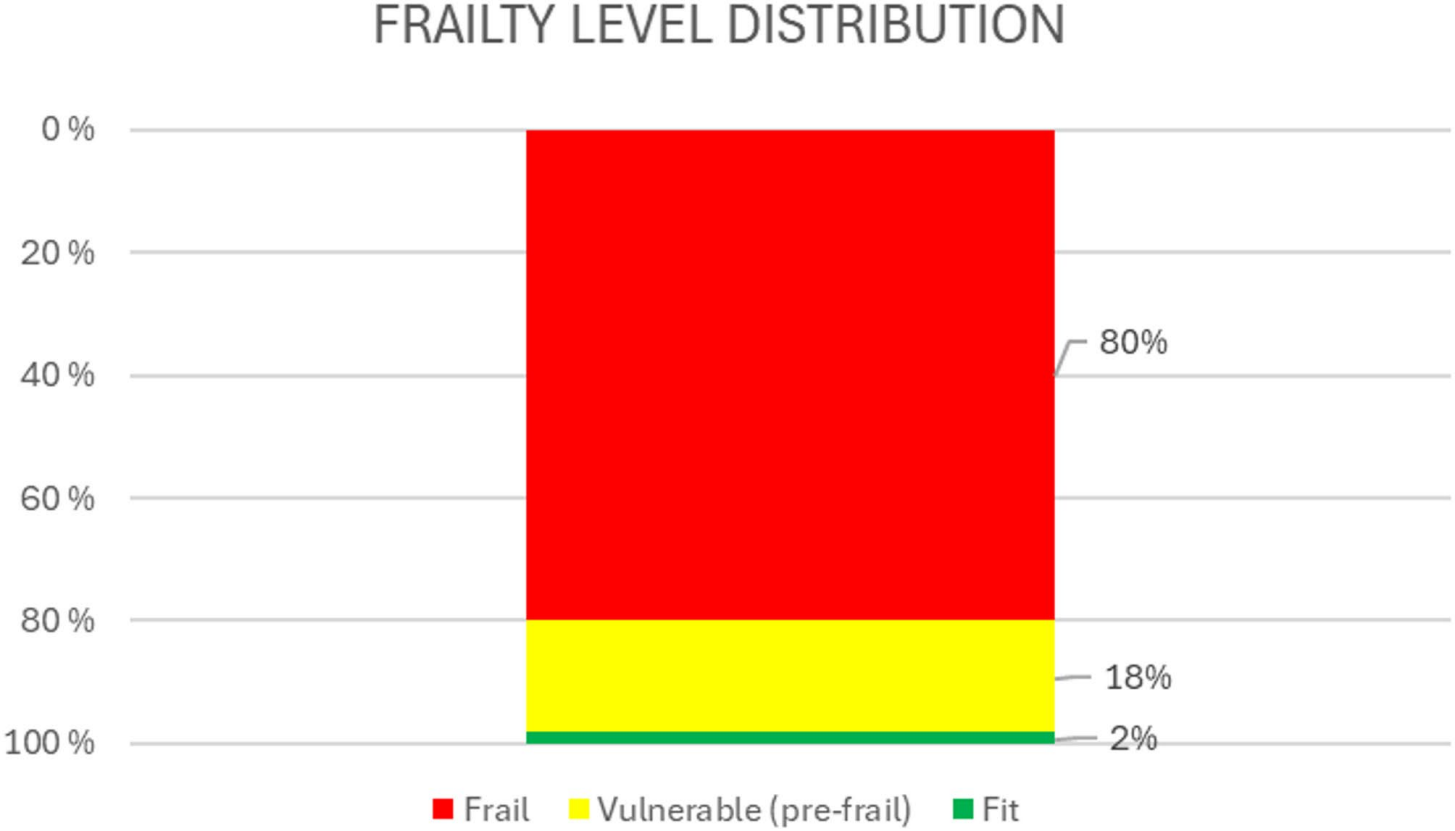
Frailty levels among the 86 home-dwelling older patients. The red and yellow bars visually underscore the prevalence of frailty and pre-frailty, highlighting areas that require attention

Figure 2 presents a traffic light bar chart categorising the patients into frail (80%, *n* = 69), pre-frail (⁓18%, *n* = 15), and fit (2%, *n* = 2) groups. This representation offers a clear overview of the health status distribution among the participants, emphasising the importance of monitoring and addressing the needs associated with these categories.

In addition to overall frailty distribution, the EFS provided insights into specific domains of Health and function, revealing key challenges within this patient population while illustrating its comprehensiveness in capturing clinically relevant frailty data in primary care. Cognitive assessments reflected significant challenges, as approximately 80% of patients (*n* = 70) failed the clock test, indicating potential cognitive impairments that require further exploration. Additionally, 66% (*n* = 57) had been admitted to hospital at least once over the past year, suggesting a correlation between frailty and increased health service utilisation.

**Fig. 3:**
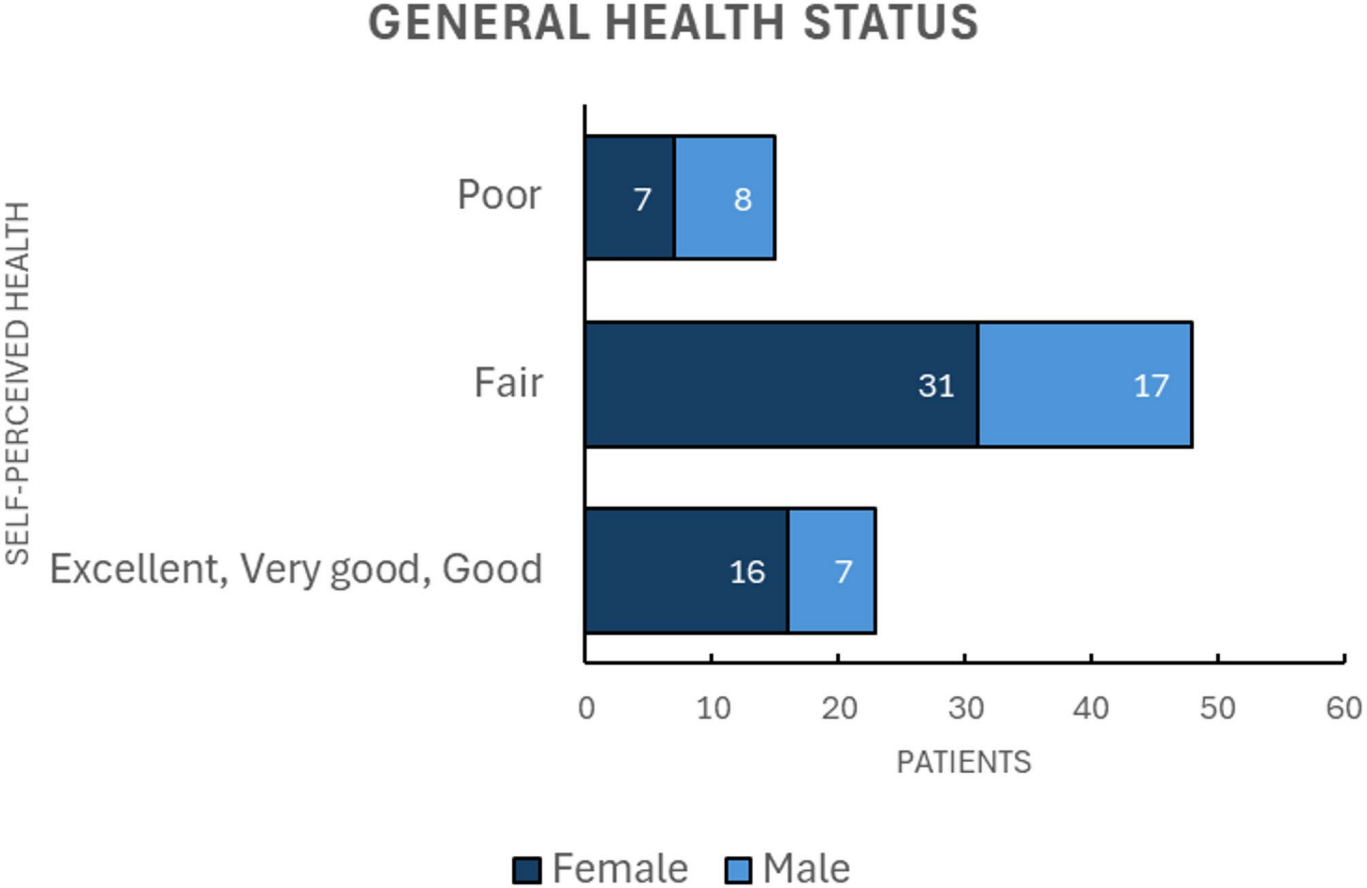
General health status among patients

In terms of self-perceived health, most participants (83%, *n* = 71) rated their Health as very good or fair, while 17% (*n* = 15) described it as poor, as depicted in Fig. 3. This self-assessment contrasts with their frailty scores, highlighting the EFS’s ability to integrate objective data with patient-reported outcomes.

Functional dependency was evident, with nearly half of the patients (*n* = 42) requiring assistance with between two and four daily activities. Social support seemed strong as well, as nearly all participants reported always (*n* = 62) or sometimes (*n* = 23) having someone to rely on for social support when needed, crucial for promoting mental and emotional health.

Medication usage was high, with about three out of four participants (78%, *n =* 67) regularly taking more than five medications and one in six (16%, *n* = 14) occasionally forgetting to take them. These results highlight the complexity of medication management in older adults and the necessity of effective monitoring and support systems. The EFS’s ability to capture such complexities demonstrates its potential for practical application in routine healthcare.

Additionally, 37% of patients (*n* = 32) had experienced recent weight loss, and 29% (*n* = 25) reported feelings of sadness or depression, signalling potential nutritional and mental health issues that require attention. Urinary incontinence affected approximately half of the participants (*n* = 44), while less than one in six participants passed the Timed Up and Go test (*n* = 13), indicating significant mobility challenges. These findings indicate that the EFS captures a wide range of interrelated health issues, providing valuable insights for understanding the needs of older adults.

Overall, these findings reflect the multifaceted nature of frailty and the complexity of health challenges among older adults with multimorbidity, underscoring the EFS’s potential as a feasible and practical tool in primary care.

## Discussion

This study investigated the feasibility of using the EFS in Norwegian primary healthcare by conducting focus group interviews with HCPs and assessing EFS frailty scores in older adults with multimorbidity. We focused on the EFS’s relevance, comprehensiveness and ease of use, and how these factors enhance multimorbidity management. The interviews offered detailed insights into the HCPs’ experiences and perspectives, while the frailty scores provided important context by characterising the patient population. Together, these data sources enabled a comprehensive evaluation of the EFS’s feasibility in primary healthcare settings.

Focus group interviews with HCPs identified three themes: The EFS enables collaborative planning, facilitates comprehensive assessments and requires proper training for effective integration. The quantitative results indicated diverse frailty scores, with a high prevalence of both pre-frailty and frailty. The EFS scores revealed cognitive challenges among the patients, increased healthcare utilisation and discrepancies between self-perceived and objective health assessments, alongside functional dependency, complex medication management, and nutritional, mental health and mobility issues. Together, our findings highlight the complexity of managing frailty in individuals with multimorbidity and suggest that the EFS may be feasible in supporting consistent assessments and tailored healthcare interventions.

### Relevance of the EFS for multimorbidity management

The EFS appears highly relevant, as the HCPs indicated that it may enhance coordination and communication among HCPs by structuring assessments, strengthening collaborative planning and aligning with multimorbidity management recommendations [9, 43]. Its relevance lies in its ability to provide a structured framework for addressing the complex health needs, ensuring consistency and clarity in multimorbidity management. By providing standardised criteria for assessing frailty, the HCPs experienced the EFS as a tool that can help them communicate using a common language and more precise discussion in multidisciplinary meetings. The HCPs found the EFS and its systematic approach helpful in terms of early recognition of health concerns, enabling them to prioritise care and thereby facilitate timely interventions.

The HCPs tested the EFS on a sample of older adults with multimorbidity, characterised by pre-frailty and frailty, as indicated by the EFS scores. This highlighted its relevance in identifying specific frailty domains, such as high medication use, recent weight loss, urinary incontinence, mobility challenges and feelings of sadness or depression, and guided targeted interventions. This aided HCPs in addressing these issues more effectively. This aligns with research suggesting that frailty assessments can prevent functional decline and improve multimorbidity management in older adults [9], with the EFS recognised as an exceptionally useful instrument [21]. By identifying frailty domains, the EFS helps HCPs collaboratively plan care tailored to individual patient needs. For instance, the multidimensional insights provided by the EFS can guide care teams in determining appropriate interventions, such as physical therapy, nutritional support or medication adjustments based on a patient’s frailty level. The relevance of the EFS lies in its ability to bridge individualised care with interdisciplinary collaboration, ensuring both interdisciplinary and patient-centred multimorbidity management [49].

The high prevalence of pre-frailty and frailty among the participating patients highlights the need for care strategies that focus on maintaining functional independence and supporting person-centred outcomes [50]. Previous research also indicates that individuals can even transition from frail to pre-frail status, underscoring the EFS’s role in effectively guiding early and targeted interventions [21, 50, 51]. The range of the patients’ EFS scores also reflects the diverse health statuses within the study population, showing an appropriate distribution without significant ceiling or floor effects, thereby reliably capturing changes in health status [52]. This capability is particularly relevant for managing older adults with multimorbidity [53], as it enables continuous monitoring and timely interventions based on frailty progression.

Our sample was characterised by pre-frailty and frailty categories, yet most patients rated their health as very good or fair, revealing a disconnect between subjective self-perception and objective assessments. Although the results were obtained by newly trained HCPs, and some scoring variability may have occurred, recent studies support this discrepancy [54], highlighting the need to consider patients’ self-perceptions, as these can influence their engagement with healthcare services and facilitate more personalised care [55]. This also underscores the importance of balancing subjective assessments with objective measures, as instruments like the EFS can guide tailored interventions that align with patients’ self-reported priorities.

### Comprehensiveness of the EFS in addressing health needs

The EFS’s comprehensiveness is evident in its multidimensional evaluations, which captures a broad range of health aspects, including cognitive function, medication use, mood and mobility challenges. This holistic assessment not only enables HCPs to gain a deeper understanding of patients’ overall health and functional levels but also supports the identification of less visible issues, such as social isolation or early nutritional deficits, that may not emerge in routine clinical consultations. Its comprehensiveness ensures that multiple dimensions of frailty are addressed simultaneously, leaving fewer gaps in patient care. One systematic review confirms the EFS’s extensive coverage of multidimensional geriatric conditions, effectively identifying areas of frailty [35].

The HCPs noted that the EFS’s multidimensional nature improved usual practice by providing timely insights into patients’ health, which would otherwise require more time to discover. This speeds up decision-making and interventions, aligning with studies that argue the value of multidimensional instruments in busy primary care settings [35, 43, 56]. This comprehensiveness enhances efficiency and provides a structured framework for addressing complex health needs. The HCPs in the study recognised the usefulness of EFS scores for tracking frailty levels and guiding service allocation. By revealing the complexity and severity of frailty, the EFS enables primary care teams to target specific components through interventions or refer patients to geriatric specialists. This dual capacity for patient monitoring and resource management is supported by research emphasising comprehensive approaches to managing frailty in individuals with multimorbidity [43, 57]. This comprehensiveness allows HCPs to integrate multiple aspects of care into a unified plan, ensuring that no critical issues are overlooked. The EFS highlights critical areas of concern, which facilitates prioritising interventions and allocating resources effectively, ensuring comprehensive care plans are developed in accordance with multimorbidity guidelines.

## Comprehensibility of the EFS for primary care use

The comprehensibility of the EFS is reflected in its adaptability and ease of use, suggesting it may serve as a practical instrument for routine practice, even in resource-constrained settings. The HCPs appreciated the potential for integrating the EFS, because it required no special equipment and was relatively simple to administer, aligning with research that advocates for practical instruments fitting seamlessly into routine practice [21]. The straightforward design of the EFS contributed to its comprehensibility, making it accessible for use by diverse healthcare teams and HCPs. The HCPs also appreciated the standardised and objective assessments provided by the EFS, which improved consistency and compatibility with existing workflows. This standardisation is crucial for managing complex conditions like frailty and multimorbidity, as supported by previous research [21, 43].

Although some HCPs questioned the reliability of self-reported answers, combining them with functional tests strengthened confidence in the EFS’s results, underscoring the importance of aligning subjective and objective health assessments for comprehensive evaluations. This balance enhanced the comprehensibility of the EFS, as it offered clarity and objectivity while accounting for patient-reported insights. The HCPs also emphasised the need for proper training to ensure effective integration of the EFS and foster confidence in the results. Specific training for scoring the clock test was considered essential to maximise its utility, given its cognitive insights and potential challenges in administration. Some HCPs considered replacing existing tests with the EFS for greater efficiency, a relevant consideration given the EFS’s demonstrated effectiveness in clinical practice [35].

Overall, while the EFS seems adaptable across various settings, the HCPs emphasised the need for its competent integration into practice, highlighting essential competencies for managing frailty in individuals with multimorbidity. The comprehensibility of the EFS further supports its feasibility in primary care, as its clear structure may simplify multimorbidity management while essential competencies ensure its effective use in tailoring interventions [21, 24]. The comprehensibility of the EFS also depends on its ethical and competent use in primary care. While frailty screening allows for early detection and person-centred care, it also carries risks such as overmedicalisation, patient anxiety, or unnecessary treatment if not paired with clear management pathways. HCPs in our study highlighted the ethical challenge of assessing frailty without the ability to follow up, which may undermine patient trust. Furthermore, the disconnect observed in our study, where patients often rated their health positively despite being classified as frail by the EFS, highlights the need for careful and empathic communication. Screening results should empower patients and foster collaborative care planning, rather than creating anxiety or undermining autonomy. These challenges align with previous findings on the variability of frailty outcomes and the importance of thoughtful use to avoid unintended consequences [58]. They also highlight the need for feasibility efforts to include training programs and resources for HCPs as well as structured follow-up frameworks to ensure that frailty screening contributes meaningfully to patient care.

Varying education levels across municipalities only underscore the necessity of adapting communication strategies to ensure effective engagement, particularly in settings with lower health literacy [59]. In addition to addressing health literacy, building a patient rapport to reduce stigma and foster trust was emphasised by several HCPs as critical for enabling open communication about sensitive issues like incontinence and mood. This approach aligns with research on the importance of patient-provider relationships in healthcare outcomes [60]. By addressing these factors, the EFS supports a patient-centred approach to frailty management in the context of multimorbidity, improving both care quality and patient engagement.

## Strengths and limitations

To our knowledge, this is the first study to explore the feasibility of using the EFS to assess frailty among home-dwelling older adults with multimorbidity in Norwegian primary care. A key strength of this study is the integration of both HCPs’ experiences and EFS frailty scores, providing a comprehensive understanding of the EFS’s feasibility and enhancing the trustworthiness of the findings. Certified EFS training ensured standardisation, though variations in scoring accuracy among HCPs remain a potential limitation. Moreover, while this study involved a sample of 86 patients and 14 HCPs, with interviews conducted with 12 of the latter, the relatively small sample sizes may limit the generalisability of the findings. The self-selection of HCPs into the study may have introduced bias, as those who chose to participate might have been more positively predisposed toward the EFS. Although the participation of HCPs from diverse settings enriched the study, the predominance of physiotherapists and limited male representation may have restricted the diversity of perspectives as well. Consequently, the findings may not be fully representative across all healthcare professions. Insights from the author team, combining familiarity with the data and external perspectives, ensured a balanced and reflexive analysis. The team’s clinical expertise and methodological experience collectively strengthened the study’s findings.

## Conclusion

Our study results indicate that the EFS is an adaptable and easy-to-use instrument that fits seamlessly into routine practice without requiring special equipment, particularly when supported by basic training for HCPs and embedded in collaborative workflows. Data from EFS scores successfully identified key health issues, such as cognitive decline and mobility challenges, highlighting why it is valuable for assessing frailty domains and broader health needs. Furthermore, interviews with HCPs revealed that the EFS facilitated standardised patient assessments and improved their communication and coordination, supporting more effective management of frailty and multimorbidity. The HCPs emphasised that the systematic approach of the EFS could enhance care planning and prioritisation of interventions. With proper training, the EFS could be seamlessly integrated into Norwegian primary healthcare workflows, particularly in settings where rapid, yet comprehensive, patient assessment is needed to support care coordination. Overall, our findings suggest that the EFS has the potential to contribute to improved care and tailored interventions for older adults with frailty and multimorbidity in Norwegian primary healthcare settings.

## Supplementary Information

Supplementary material 1.

## Data Availability

The datasets generated and analysed during the current study are not publicly available due to terms of data collection approval.

## Abbreviations

EFS: Edmonton Frail Scale
HCPs: Healthcare professionals
