# Supplementary material for: The Edmonton frail scale: a feasibility study on assessing frailty among older adults with multimorbidity in Norwegian primary health care
Source: BMC Prim Care. 2025 Sep 26;26:289. doi: 10.1186/s12875-025-02996-7 (PMC12465194; doi:10.1186/s12875-025-02996-7)
Supplement: Supplementary file 1 — Supplementary material 1. [file 12875_2025_2996_MOESM1_ESM.docx]

| **ENTRY QUESTIONS** |
| --- |
| 1. As a healthcare professional who has tested EFS on older people with multimorbidity, please share your initial impressions and experiences of using the instrument in your practice.  - Could you describe any challenges you encountered and how you addressed them? - What strategies were effective in overcoming these challenges? - Were there any specific insights or surprises that emerged during its use? |
| **PERCEIVED ACCEPTABILITY** |
| 1. How acceptable do you find the EFS among patients, regarding their willingness to participate and comfort during the assessment process?  - Could you share any feedback or reactions you received from patients? - How do patients' attitudes towards being assessed with the EFS compare to other methods? - Have patients expressed any questions or concerns? |
| **PRACTICAL MATTERS** |
| 1. Based on your experiences, what are the practical implications of using the EFS in your workflow?  - Consider assessments related to time spent, ease of administration, and integration into existing documentation routines. - What challenges have you encountered in using EFS? - Have you made any adjustments to its use? If so, what where they? - What are the key factors for the successful implementation? |
| **INTEGRATION INTO THE SERVICE** |
| 1. When reviewing the EFS with your team, what outcomes or benefits did you observe?  - Which areas were most important to patients? - How does the EFS complement or improve the assessment of frailty compared to other tools? - Are there particular areas covered by EFS that you find valuable or unique in managing older patients with multimorbidity? - Have you identified any overlap with existing assessment instruments, and how did you manage these? |
| **FURTHER USE OF THE EFS** |
| 1. What resources do you think are needed to ensure the continued use of the EFS?  - From your perspective, what is necessary for the EFS to be adopted for mainstream use? - Do you think the EFS should be used routinely in the future? |
| **ENDING QUESTION** |
| 1. Are there any other important aspects that we haven’t discussed? Is there anything else you would like to add? |
